# Obstructive sleep apnea related to mental health, health-related quality of life and multimorbidity: A nationwide survey of a representative sample in Republic of Korea

**DOI:** 10.1371/journal.pone.0287182

**Published:** 2023-06-15

**Authors:** Mee-Ri Lee, Sung Min Jung

**Affiliations:** 1 Department of Preventive Medicine, Soonchunhyang University College of Medicine, Cheonan-si, Chungcheongnam-do, Republic of Korea; 2 Department of Surgery, Inje University, Ilsan Paik Hospital, Goyang-si, Republic of Korea; University of Toronto, CANADA

## Abstract

**Objectives:**

This study assessed the effects of obstructive sleep apnea (OSA) on mental health, health-related quality of life (HRQoL), and multimorbidity in Korean adults.

**Methods:**

The study included 8030 participants from the Korea National Health and Nutrition Examination Survey Ⅷ (2019–2020). The risk of OSA was assessed using STOP-BANG questionnaire. Depression was measured using the Patient Health Questionnaire-9 (PHQ-9), and stress was measured using a questionnaire. HRQoL was determined by EuroQol 5-dimension (EQ-5D) and Health-related Quality of Life Instrument with 8 Items (HINT-8) scores. Multimorbidity was defined as the presence of 2 or more chronic diseases. A complex sample multivariate logistic regression analysis was conducted.

**Results:**

Participants with a high OSA risk were more likely to a have high PHQ-9 score (OR 4.31, 95% confidence interval [CI] 2.80–6.65), total depression (OR 4.07, 95% CI 2.67–6.19) stress (OR 2.33, 95% CI 1.85–2.95), lower EQ-5D (OR 2.88, 95% CI 2.00–4.15) and HINT-8 scores (OR 2.87, 95% CI 1.65–4.98), and multimorbidity (OR 2.62, 95% CI 2.01–3.41) than participants with low OSA risk. High OSA risk was significantly associated with all EQ-5D and HINT-8 items.

**Conclusions:**

This study adds to the few population-based studies showing associations between mental health, HRQoL, and multimorbidity using nationwide data. OSA prevention might be helpful for good mental health, improving HRQoL, and comorbidity burdens. The results provide novel insights regarding the association between sleep apnea and multimorbidity.

## Introduction

Obstructive sleep apnea (OSA) is a chronic condition characterized by frequent partial or complete obstruction of the upper airway during sleep. These events, along with the simultaneous activation of the sympathetic nervous system, cause intermittent hypoxia, hypercapnia, and recurrent arousals, resulting in sleep fragmentation [1]. Nearly 1 billion adults are affected by obstructive sleep apnea, and globally, 425 million adults aged 30–69 years have moderate to severe obstructive sleep apnea, and treatment is recommended [2]. The apnea-hypopnea index (AHI) has been most common measurement of OSA frequency and severity; it is the number of apnea and hypopneas counted per hour during sleep [3] and OSA severity is classified as mild (AHI 5.0–14.9 events/h), moderate (AHI 15.0–29.9 events/h), or severe (AHI > 30.0 event/h) [4]. OSA has complications such as hypertension, stroke, obesity, and cardiovascular disease [5].

Polysomnography (PSG) is considered the gold standard for diagnosing OSA [6]. However, there are easier to use risk assessment questionnaires for identifying high OSA risk, such as STOP-BANG, and Berlin questionnaires, and the American Society of Anesthesiologists checklist [7]. STOP-BANG questionnaire consists of 8 items: snoring, tiredness, observed apnea, high blood pressure, body mass index (BMI), age, neck circumference, and male sex [8]. A recent review found that the STOP-BANG questionnaire (score cutoff ≥ 3) presented the highest sensitivity (92%–96%) for detecting OSA but lacked specificity (27%–35%) in both sleep clinic and surgical populations [9].

In the Republic of Korea, 19.8% of participants involved in general health checkups in 2020 had mild to severe depression symptoms [10]. Recently, mental health in the general population was the worst from before coronavirus disease 2019 (COVID-19) to during COVID-19, suicidal planning depression, and severe stress increased significantly in men [11]. A recent Korean study showed that poor sleep quality is associated with lower quality of life [12]. Although there have been some studies on the link between OSA and depression [13] or health-related quality of life (HRQoL) [14, 15], the results of previous studies have been conflicting and an insufficient number of studies have been performed in the Korean population. The influence of OSA on cardiovascular disease, especially hypertension, has been studied [16], however there are few articles on the relationship between sleep apnea and multimorbidity in the general population.

No previous studies have assessed the relationship between OSA and multimorbidity in the general population using the STOP-BANG questionnaire. Therefore, this study evaluated the relationship between OSA and mental health, HRQoL, and multimorbidity using data from the Korea National Health and Nutrition Examination Survey (KNHANES) 2019–2020.

## Methods

### Study design

**Fig 1:**
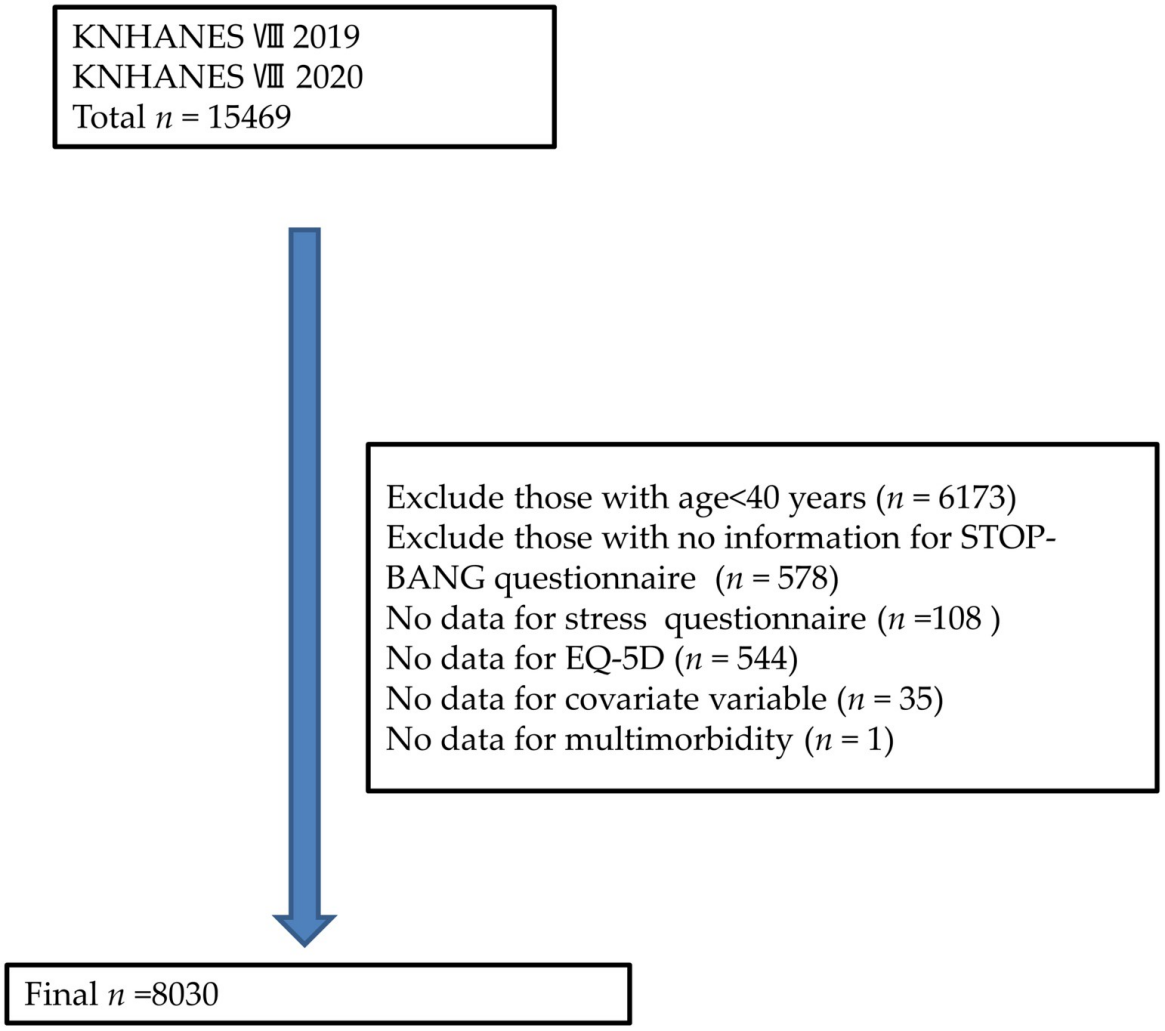
Flow chart for the number of participants excluded and analyzed. Abbreviations: KNHANES, Korea National Health and Nutrition Examination Survey; EQ-5D: EuroQol 5-dimension.

Combined KNHANES VIII (2019–2020) data were used in this study. The KNHANES is a national cross-sectional survey of the population conducted annually by the Centers for Disease Control and Prevention, using a complex sample survey with a multistage sampling method. A total of 15,469 people participated in the KNHANES, and the STOP-BANG questionnaire was administered to individuals aged ≥ 40 years. We excluded participants who were younger than 40 years old, and those who had no information on the STOP-BANG questionnaire, or stress questionnaire. The final number of participants analyzed in this study was 8030 (Fig 1).

### Institutional review board statement

This study approved by the Institutional Review Board of Inje University Ilsan Paik Hospital (ISPAIK 2022-11-008).

### STOP-BANG score

The risk of obstructive sleep apnea was determined using the STOP-BANG score. The STOP-BANG score ranges from 0 to 8 by adding 1 point to each of the 8 questionnaires according to yes/no: (1) Snoring: Is your snoring louder than the conversation or loud enough to be heard in the next room? (2) Tiredness: Do you often feel tired or sleepy during the day? (3) Observed Apnea: Has anyone seen you stop breathing when you sleep? (4) Pressure: High blood pressure is defined as measuring 140/90 mmHg or higher or taking hypertension medication. (5) BMI: Physicians measure weight and height and calculate a BMI higher than 35. (6) Age: Are you over 50 years? (7) Neck Circumference: Physicians measure neck circumference and measurements greater than 40 cm. (8) Sex: Are you male?

We divided the participants into 3 groups (low risk, intermediate risk, and high-risk of OSA) according to the STOP-BANG classification guidelines [17]. An answer of yes to 0–2 questions indicated low risk, 3–4 questions indicated intermediate risk, and 5–8 questions indicated high risk. Those with 2 or more yes answers for the 4 STOP questions and at least 1 condition (male, BMI > 35 kg/m^2^, neck circumference > 40 cm) were classified in the high-risk group.

### Depression and stress

For major depressive disorder screening, the Patient Health Questionnaire-9 (PHQ-9) was administered in 2020. The PHQ-9 has 9 items that match the Diagnostic and Statistical Manual of Mental Disorders, 4th Edition criteria as 0 to 3 (0 never; 1 several days; 2 more than a week; and 3 almost every day) [18]. The summed PHQ-9 item score simply adds up each item’s scores to give a total score between 0 and 27. The PHQ-9 has been translated into Korean and can be downloaded free from the PHQ website [19]. The criteria for a depressive disorder using the Korean version of the PHQ-9 is a score of 5 [20].

Depression by questionnaire was defined as someone who has answered that they are currently suffering from it or receiving treatment it. Total depression was defined as having a PHQ-9 score of 5 or higher, answering that they are currently suffering from depression, or receiving treatment for depression.

Stress was measured using the questionnaire “How much stress do you feel in your daily life?” The answers to the questionnaire were as follows: I feel very much, I feel a lot, I feel a little, and I do not feel very much. Those who responded that they tend to feel “very much” or “a lot” of stress in their daily life were defined as being stressed.

### HRQoL

HRQoL was assessed using the EuroQol 5-dimension (EQ-5D) instrument and Health-related Quality of Life Instrument with 8 Items (HINT-8). The EQ-5D consists of 5 items, and the details of EQ-5D have been previously described [21]. For the analysis of each of the 5 items of the EQ-5D, 3 levels were divided into 2 groups: “no problem at all” was designated to the “no” group, “some problem” or “many problems” were designated to the “having a problem” group.

The HINT-8 consists of 8 items and 4 responses; the range was 0.132 (worst) to 1 (best). The details of the HINT-8 have been described in a previous article and on the KNHANES website [22, 23]. To analyze each item of the HINT-8 we divided the answers into 2 groups: “no” was designated to the “no problem” group and “mild,” “moderate,” and “severe” were designated to the “having problem” group. The HINT-8 was administered only in 2019.

### Multimorbidity

Twenty-five chronic diseases from the KNHANES questionnaires were counted and multimorbidity was defined as 2 of more confirmed chronic diseases. Chronic disease includes dyslipidemia, stroke, myocardial infarction, angina, osteoarthritis, rheumatic arthritis, tuberculosis, asthma, allergic rhinitis, depression, kidney failure, atopic dermatitis, diabetes mellitus, thyroid disease, stomach cancer, liver cancer, colon cancer, breast cancer, cervical cancer, lung cancer, thyroid cancer, liver cirrhosis, hepatitis B, and hepatitis C. The details for determining multimorbidity was as previously described [24].

### Covariates

Education, smoking status, alcohol drinker, and physical activity were investigated using a self-report questionnaire. Education had 3 levels (low, less than 9 years; medium, 10–12 years; and high, 13 years or more). Smoking status was classified as lifetime non-smokers, former smokers (those who smoked in the past but no longer smoke), and current smokers (those who have smoked 100 or more cigarettes in their lifetime and continue to smoke). Alcohol drinkers were defined as those who have consumed 1 or more drinks per month in the past year. Physical activity was defined as moderate-intensity activity of 150 minutes or more per week, high-intensity activity of 75 minutes or more per week, or a combination of moderate and high-intensity physical activity.

Body mass index (BMI) was calculated as weight divided by height squared (kg/m^2^).

Hypertension is defined as having a systolic blood pressure of 140 mmHg or higher, a diastolic blood pressure of 90 mmHg or higher, or taking medication for high blood pressure. Diabetes is defined as having a fasting blood glucose level of 126 mg/dL or higher, taking diabetes medication or insulin injections, being diagnosed by a doctor, or having a hemoglobin A1c level of 6.5% or higher. Cardiovascular disease was defined as a person who is being treated for myocardial infarction or angina.

### Statistical analyses

Analyses were performed using a complex sample analysis, according to the statistical guidelines provided by the Centers for Disease Control and Prevention.

The chi-square test or one-way analysis of variance was used for group comparisons of general characteristics according to OSA risk.

Those with EQ-5D or HINT-8 scores of < 10% were defined as having a low HRQoL.

The Cochran-Armitage test for trend was used to determine the weighted prevalence of mental health, HRQoL, and multimorbidity according to OSA risk and the trend for an increasing prevalence.

Multivariate logistic regression for depression and HRQoL by HINT-8 was adjusted by age, sex, education, alcohol consumption, smoking, physical activity, BMI, hypertension, diabetes, cardiovascular disease. We used a complex sample multivariate logistic regression adjusted for age, sex, education, alcohol consumption, smoking, physical activity, year (2019, 2020), BMI, hypertension, diabetes, cardiovascular disease to calculate the odds ratios (OR) with 95% confidence intervals (CIs) for stress and HRQoL by EQ-5D. And multivariate logistic regression for multimorbidity was adjusted for age, sex, education, alcohol consumption, smoking, physical activity, year (2019, 2020), and BMI.

The *p* value was significant for 0.05 or less, 2-sided. All statistical analyses were performed using Stata version 17 (Stata Corp., College Station, TX, USA) and graph was used by the R software, version 4.2.2 (The Comprehensive R Archive Network: http://cran.r-project.org, accessed August 30, 2022).

## Results

The participants’ general characteristics according to OSA risk are shown in Table 1. The high and intermediate OSA risk groups had more men, current smokers, consumers of alcohol, stress, depression, multimorbidity, higher education level, diabetes, hypertension, cardiovascular disease and lower HRQoL scores, than the low-risk group.

**Table 1:** Participants’ sociodemographic and clinical characteristics according to risk of obstructive sleep apnea.

Variable	Low	Intermediate	High	Total	*P* value
n	4857	2038	1135	8030	
Age, y,	57.3 (11.7)	65.0 (9.56)	59.3 (11.2)	59.5 (11.6)	**<0.001**
Women	3662 (69.9)	847 (36.7)	38(2.9)	4547 (51.6)	**<0.001**
Year					
2020	2278(49.2)	966(48.7)	538(50.7)	3782(49.4)	0.645
Education					
Low	1504 (23.9)	941 (39.3)	298 (19.1)	2743 (26.7)	**<0.001**
Medium	1576 (35.4)	630 (33.3)	369 (33.4)	2575 (34.6)	
High	1777 (40.7)	467 (27.5)	468 (47.5)	2712 (38.7)	
Smoking status					
Never smoker	3583(69.9)	1000(45.2)	216(18.8)	4799(56.1)	**<0.001**
Former smoker	745(16.9)	690(35.2)	577(49.1)	2012(26.3)	
Current smoker	529(13.1)	348(19.6)	342(32.1)	1219(17.7)	
Alcohol consumption					
Alcohol drinker	2031(45.5)	1014 (53.6)	777 (69.9)	3822 (51.2)	**<0.001**
Physical activity					
Yes	1900 (40.2)	769 (39.6)	473 (40.0)	3142 (40.0)	0.930
Stress					
Yes	1090 (22.9)	449 (22.4)	331 (32.2)	1870 (24.3)	**<0.001**
Depression by PHQ-9 (n = 3748)					
Yes	302 (13.4)	183 (16.7)	101 (19.5)	586 (15.2)	**0.003**
Total depression (n = 3748)					
Yes	327(14.4)	197(17.6)	104(20.1)	628(16.1)	**0.008**
HRQoL					
EQ-5D (n = 8030)	351 (5.7)	340 (13.5)	125(7.8)	816 (7.8)	**<0.001**
HINT-8 (n = 4236)	183 (5.6)	155 (12.4)	55 (7.1)	393 (7.5)	**<0.001**
Multimorbidity	855 (14.8)	653 (28.7)	275 (22.2)	1783(19.2)	**<0.001**
Hypertension	1107(20.1)	1440(67.1)	798(66.7)	3345(38.4)	**<0.001**
Diabetes	838(15.8)	642(29.0)	376(30.8)	1856(21.2)	**<0.001**
CVD	109 (1.8)	109(4.5)	64(4.6)	282(2.9)	**<0.001**

Data were presented as mean (standard deviation) or number (percentage). χ^2^ test and one-way ANOVA were used for categorical and continuous variables, respectively.

Bold numbers highlight the statistical significance.

Abbreviations: CVD, cardiovascular disease; HINT-8, Health-related Quality of Life Instrument with 8 Items; HRQoL, health–related quality of life; EQ-5D, EuroQol 5-dimension; PHQ-9, Patient Health Questionnaire-9

**Fig 2:**
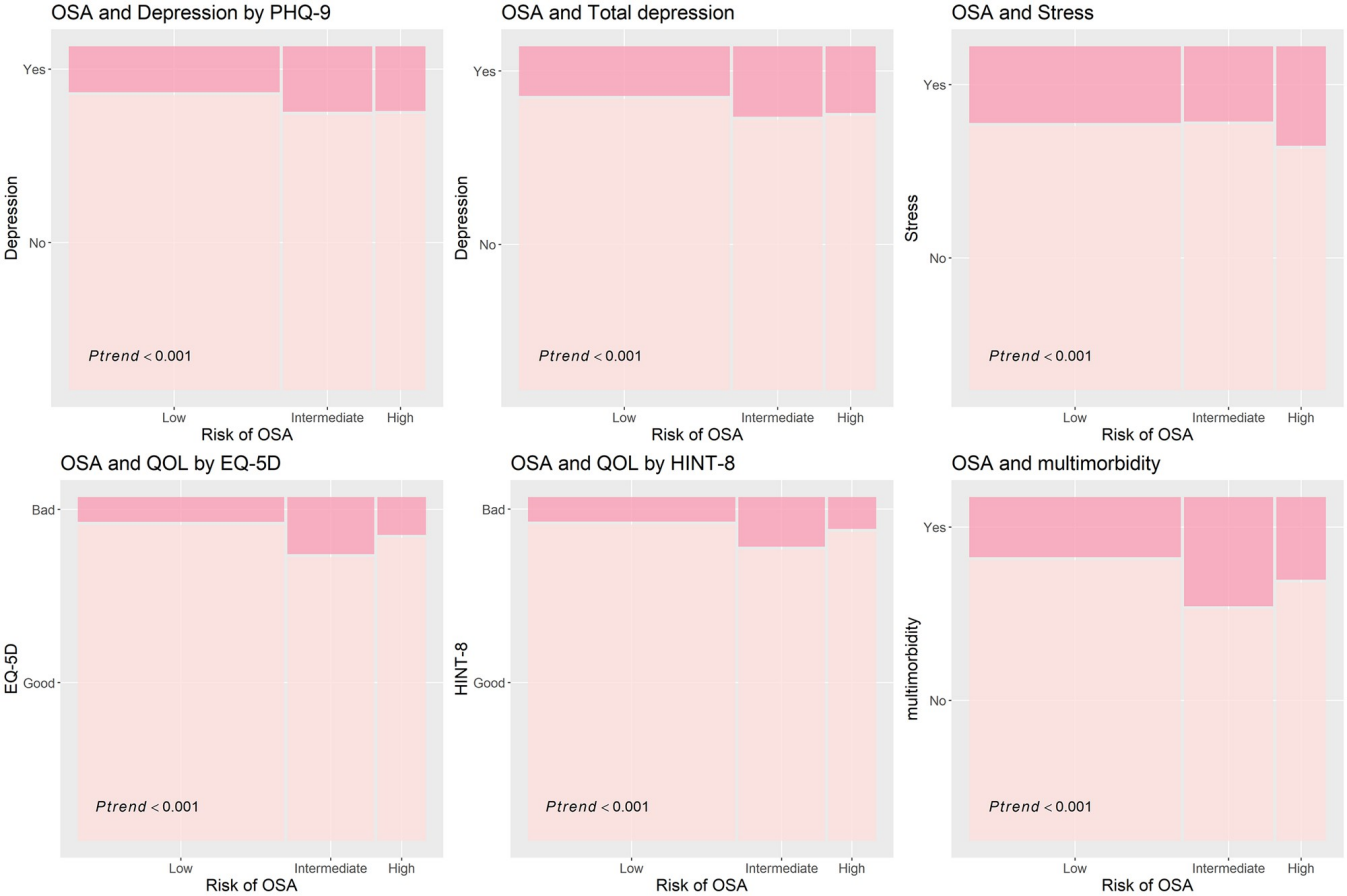
Prevalence of depression, stress, low EQ-5D and low HINT-8 scores, and multimorbidity according to OSA risk. Abbreviations: EQ-5D, EuroQol 5-dimension; HINT-8, Health-related Quality of Life Instrument with 8 Items; OSA, obstructive sleep apnea; PHQ-9, Patient Health Questionnaire-9; QOL, quality of life.

The estimated prevalence of depression by PHQ-9, total depression, stress, low HRQoL per the EQ-5D and HINT-8 scores, and multimorbidity in the high OSA risk group were 18.9%, 19.5%, 29.1%, 11.0%, 9.2%, and 24.2%, respectively. The trends for the prevalence of depression by PHQ-9, total depression, stress, HRQoL per the EQ-5D and HINT-8 scores, and multimorbidity significantly increased according to OSA risk (Fig 2).

**Fig 3:**
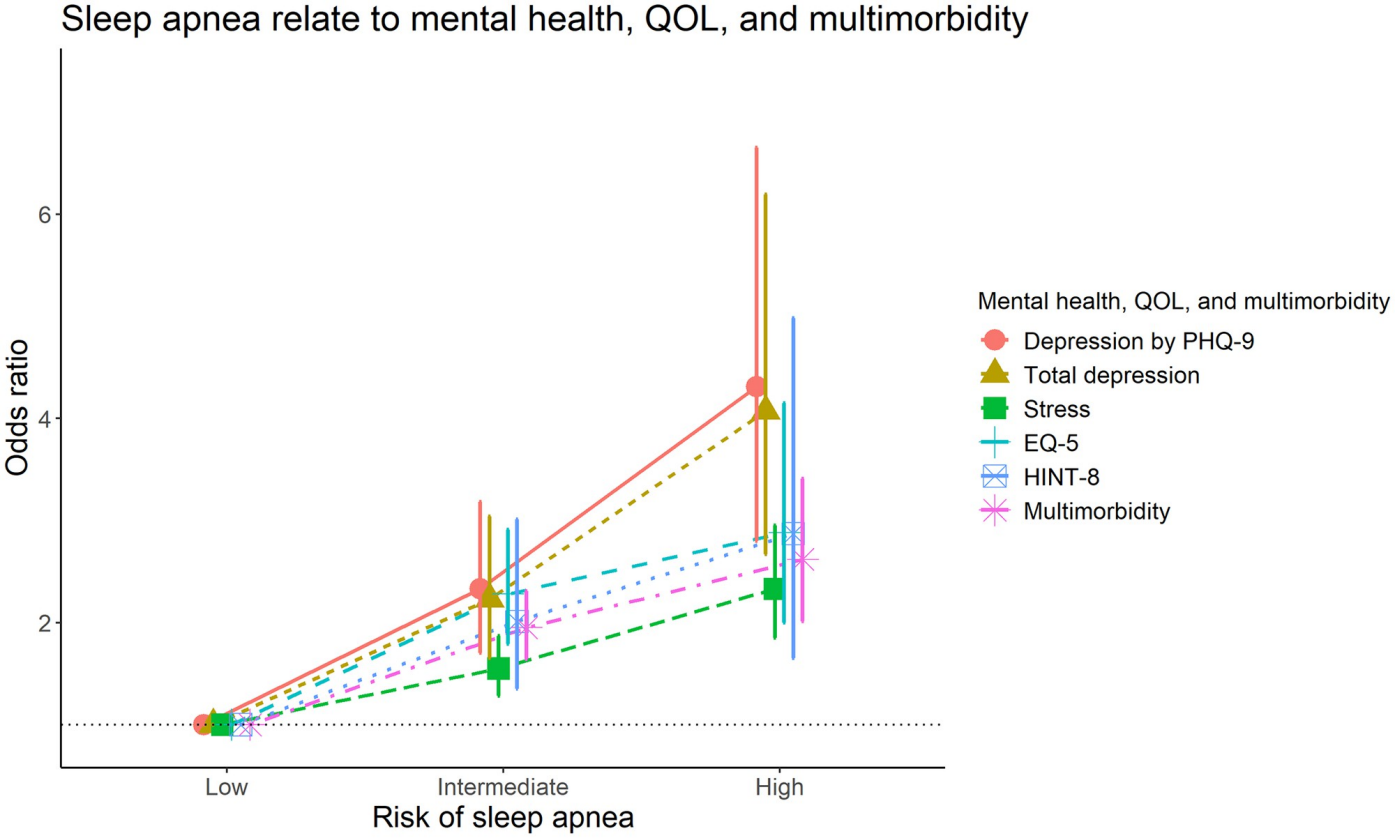
Association between sleep apnea risk and mental health, quality of life based on EQ-5 and HINT-8 scores, and multimorbidity using a multivariate logistic regression analysis. Age, sex, education, alcohol consumption, smoking status, and physical activity, year, BMI, hypertension, diabetes, and cardiovascular disease were adjusted when calculating odds ratio for depression and QOL by HINT-8. Age, sex, education, alcohol consumption, smoking status, and physical activity, year, BMI, hypertension, diabetes, and cardiovascular disease were adjusted when calculating odds ratio for stress and quality of life by EQ-5D, while age, sex, education, alcohol consumption, smoking status, and physical activity, year and BMI were adjusted when calculating odds ratio for multimorbidity. Abbreviations: QOL, quality of life; PHQ-9, Patient Health Questionnaire-9; EQ-5D, EuroQol Five-Dimension Questionnaire; HINT-8, Health-related Quality of Life Instrument with 8 Items.

In the fully adjusted model, the OR for a higher PHQ-9 score and total depression in the high OSA risk group was 4.31 (95% CI 2.80–6.65) and 4.07 (95% CI 2.67–6.19), respectively (Fig 3). OR for stress in the high OSA risk group was 2.33(1.85–2.95) compared with low OSA risk group. (Fig3) Considering the high OSA risk, the OR for lower EQ-5D scores was 2.88 (95% CI 2.00–4.15) and lower HINT-8 scores was 2.87 (95% CI 1.65–4.98) compared to participants with a low OSA risk (Fig 3). For higher OSA risk, the OR of having multimorbidity was 2.62 with significance (95% CI 2.01–3.41) compared with low OSA risk (Fig 3).

The ORs for the relationship between the risk of sleep apnea and the 5 items of EQ-5D and 8 items of HINT-8 using a multivariate logistic regression are presented in Table 2. The risk of sleep apnea was significantly related to all items of the EQ-5D and HINT-8 (Table 2).

**Table 2:** The association between risk of sleep apnea and each items of EQ-5D and HINT-8.

	Event	Low	Intermediate	High
		Reference	OR (95%CI)	OR (95%CI)
EQ-5D (n = 8030)				
Problem of Mobility	1377	1	**1.71(1.39–2.09)**	**2.26(1.68–3.05)**
Problem of Self-care	350	1	**1.89(1.33–2.68)**	**2.47(1.46–4.16)**
Problem of usual activities	670	1	**2.12(1.63–2.75)**	**2.71(1.86–3.94)**
Having pain/discomfort	1964	1	**1.67(1.39–2.00)**	**2.18(1.69–2.81)**
Having anxiety/depression	790	1	**2.04(1.55–2.68)**	**3.13(2.11–4.64)**
HINT-8 (n = 4236)				
Problem of Climbing stairs	452	1	**2.27(1.60–3.21)**	**2.96(1.75–5.00)**
Having pain	400	1	**2.05(1.49–2.83)**	**3.07(1.70–5.53)**
Problem of vitality	1496	1	1.22(0.97–1.53)	**1.41(1.02–1.95)**
Problem of working	419	1	**2.34(1.57–3.49)**	**2.52(1.56–4.07)**
Having depression	222	1	**1.90(1.20–2.99)**	**1.99(1.01–3.92)**
Problem of memory	223	1	**2.14(1.31–3.50)**	**3.92(2.13–7.18)**
Problem of sleep	435	1	1.36(0.96–1.91)	**2.16(1.34–3.49)**
Problem of happiness	1799	1	**1.24(1.00–1.54)**	**1.36(1.00–1.83)**

Bold numbers highlight the statistical significance.

Abbreviations: CI, confidence interval; EQ-5D, EuroQol Five-Dimension Questionnaire; HINT-8, Health-related Quality of Life Instrument with 8 Items; OR, odds ratio

In subgroup analyses according to age group (middle-aged and older participants), OSA risk was associated with mental health, HRQoL, and multimorbidity except QOL by HINT-8 for middle-aged (Table 3). In subgroup analyses according to sex, the increased risk of OSA was linked to depression, stress, low level of HRQoL by EQ-5D, and greater likelihood of multimorbidity in both men and women and women and OR was higher in women than in men (Table 4).

**Table 3:** The association between risk of sleep apnea and mental health, quality of life, and multimordibity by age group using multivariate logistic regression.

	< 65				≥ 65			
	Event (N)	Low	Intermediate	High	Event (N)	Low	Intermediate	High
		Ref.	OR (95%CI)	OR (95%CI)		Ref.	OR (95%CI)	OR (95%CI)
Depression by PHQ-9 (n = 3748)	381 (2460)	1	**2.19(1.40–3.41)**	**4.32(2.57–7.25)**	205 (1288)	1	**3.05(1.88–4.95)**	**4.91(2.27–10.63)**
Total depression (n = 3748)	402 (2460)	1	**2.07(1.35–3.18)**	**4.06(2.45–6.74)**	226 (1288)	1	**2.98(1.89–4.69)**	**4.60(2.21–9.60)**
Stress (n = 8030)	1407 (5225)	1	**1.48(1.17–1.87)**	**2.30(1.77–2.98)**	463 (2805)	1	**2.11(1.54–2.89)**	**2.83(1.714.672)**
QOL by EQ-5D (n = 8030)	258 (5225)	1	**1.93(1.25–2.98)**	**2.94(1.68–5.14)**	558 (2805)	1	**2.68(1.97–3.63)**	**3.00(1.87–4.82)**
QOL by HINT-8 (n = 4236)	130 (2748)	1	1.61(0.81–3.21)	2.15(0.85–5.41)	1488 (263)	1	**2.89(1.82–4.59)**	**4.56(2.43–8.52)**
Multimorbidity (n = 8030)	755 (5225)	1	**1.94(1.50–2.53)**	**2.77(1.93–3.97)**	1028 (2805)	1	**1.80 (1.44–2.26)**	**2.24 (1.58–3.17)**

Bold numbers highlight the statistical significance.

Abbreviations: CI, confidence interval; HINT-8, Health-related Quality of Life Instrument with 8 Items; EQ-5D, EuroQol 5-dimension; OR, odds ratio; PHQ-9, Patient Health Questionnaire-9; QOL, quality of life

**Table 4:** The association between risk of sleep apnea and mental health, quality of life, and multimordibity by sex group using multivariate logistic regression.

	Men				Women			
	Event (N)	Low	Intermediate	High	Event (N)	Low	Intermediate	High
		Ref.	OR (95%CI)	OR (95%CI)		Ref.	OR (95%CI)	OR (95%CI)
Depression by PHQ-9 (n = 3748)	189 (1659)	1	1.39(0.83–2.34)	**2.84(1.72–4.70)**	397 (2089)	1	**3.35(2.36–4.75)**	**10.62(3.93–28.71)**
Total depression (n = 3748)	200 (1659)	1	1.28(0.78–2.12)	**2.69(1.67–4.34)**	428 (2089)	1	**3.29(2.34–4.61)**	**8.82(3.25–23.96)**
Stress (n = 8030)	740 (3483)	1	**1.39(1.05–1.84)**	**2.19(1.66–2.88)**	1130 (4547)	1	**1.96(1.55–2.50)**	**4.65(2.17–9.96)**
QOL by EQ-5D (n = 8030)	279 (3483)	1	**2.72(1.71–4.33)**	**3.64(2.19–6.04)**	537 (4547)	1	**2.10(1.59–2.76)**	**2.80(1.10–7.13)**
QOL by HINT-8 (n = 4236)	114 (1805)	1	1.19(0.45–3.16)	2.08(0.83–5.17)	279 (2431)	1	**2.33(1.60–3.40)**	**5.58(1.05–29.70)**
Multimorbidity (n = 8030)	640 (3483)	1	**1.65(1.24–2.18)**	**2.33(1.71–3.18)**	1143 (4547)	1	**2.17 (1.77–2.67)**	2.32(0.96–5.60)

Bold numbers highlight the statistical significance.

Abbreviations: CI, confidence interval; HINT-8, Health-related Quality of Life Instrument with 8 Items; EQ-5D, EuroQol 5-dimension; OR, odds ratio; PHQ-9, Patient Health Questionnaire-9; QOL, quality of life

In sensitivity analysis, where multimorbidity was additionally adjusted, the increased risk of OSA remained significantly associated with poorer mental health and lower quality of life (QOL) (S1 Fig).

## Discussion

This cross-sectional study demonstrated an association between OSA risk and depression, stress, HRQoL, and components of HRQoL in adults aged 40 years or older. To the best of our knowledge, this is the first study to show the relationship between sleep apnea, using a reliable and easy-to-use tool, the STOP-BANG questionnaire, and multimorbidity from a nationwide survey data of a representative sample.

Our finding of an association between OSA and mental health is consistent with other studies [25–28]. In the US population, men who snore or stop breathing more than 5 nights/week have the strongest association with probable major depression [27]. A previous study showed that a high OSA risk determined by the STOP-BANG questionnaire was more often diagnosed with depression than with low risk for 16,448 in South Carolina, United States [26]. Psychological stress was high in patients with OSA, and the presence of OSA, not severity, and was related to depression and anxiety in 44 untreated participants with OSA and 57 health controls in a study in the United States [28]. OSA prevalence may increase in patients with major depressive disorder (MDD) and posttraumatic stress disorder [25].

A study by Kang et al. [29] revealed that OSA might affect insular neuron damage or dysfunction, which correlated with the Hamilton Depression Scale. Another possibility is that the shared link between OSA and depression may lead to sleep disturbances, possibly overlapping the symptoms [13]. Obesity is also a risk factor for OSA [30] and depression [31]. The final possible contributing factors could be chronic inflammation and oxidative stress, which significantly correlate with OSA [32] and are higher in people with MDD [33].

The results of the current study showed an association between obstructive sleep apnea and HRQoL and almost all components of HRQoL, which are consistent with a previous study [34–37]. In US randomized controlled trial, continuous positive airway pressure, which was the treatment, for 483 patients with severe OSA and used ≥ 4 h per night, quality of life improved as measured by the Calgary Sleep Apnea Quality of Life Index [34]. For 395 urban Georgian participants, physical component summaries from the Short Form Health Survey 12 (SF-12) which measures HRQoL, were significantly lower among those with a sleep disorder [35]. In contrast, a cohort study of 4420 Pomerania German participants demonstrated that AHI severity was related to mental component summaries of the SF-12, although the association between AHI and physical components of QOL was not verifiable [37]. A recent systematic review demonstrated that positive airway pressure was associated with a significant improvement in HRQoL measured by the 36-Item Short Form Health Survey mental and physical health components [36].

For 745 men over 40 years old in South Australia, the presence of OSA was associated with multimorbidity [38]. A Canadian study of 120 patients presenting with OSA revealed that severe OSA was related to severity of multimorbidity using the Disease Burden Morbidity Assessment [39]. In a Finnish study, sleep apnea was related with depression and 63% of the individuals with sleep apnea were multimorbid at their sleep apnea diagnosis, compared with 35% in the general population, and 34% were severely multimorbid with 4 or more chronic diseases compared to 14% of the general population [40].

The main strength of the present study was that it was the first to demonstrate a highly significant link between OSA risk using STOP-BANG scores and multimorbidity in a large nationally representative general population. We used various mental health and HRQoL measurements.

Despite the strengths of our study, it has several limitations. One limitation is cross-sectional data was used. Therefore, causal relationships between OSA and mental health and between OSA and HRQoL could not be determined. Several studies have shown a bidirectional relationship. Second, because the STOP-BANG questionnaire was only available for accessing OSA, we could not cross-validate other measurements, such as the gold-standard PSG or oxygen desaturation index. Although the STOP-BANG questionnaire has limitations in determining OSA, a recent study showed that it was the most useful screening tool with acceptable sensitivity and specificity (72.4 and 67.8%, respectively) and area under the receiver-operator characteristic curve (0.75) for moderate to severe OSA in the Korean population [41]. Third, we did not evaluate the use of positive airway pressure. Fourth, we don’t have information about medication for obstructive sleep apnea. Fifth, KNHANES data do not contain information about participants’ SARS-Cov-2 infection that could affect their mental health or quality of life.

## Conclusion

This study found that in a general Korean population, a high OSA risk was closely associated with poor mental health and HRQoL, and multimorbidity compared to a low OSA risk. It is important to investigate the factors contributing to poor mental health and HRQoL, and multimorbidity in OSA. More detailed research should be conducted to determine the causal relationships between OSA and mental health, HRQoL, and multimorbidity.

## Supporting information

S1 Fig Relationship between obstructive sleep apnea and mental health and QOL by additionally adjusted for multimorbidity. Adjusted for age, sex, education, alcohol consumption, smoking status, and physical activity, year, BMI, and multimorbidity. Abbreviations: QOL, quality of life; PHQ-9, Patient Health Questionnaire-9; EQ-5D, EuroQol Five-Dimension Questionnaire; HINT-8, Health-related Quality of Life Instrument with 8 Items.
(TIF)
