# Supplementary figures and images for: Obstructive sleep apnea related to mental health, health-related quality of life and multimorbidity: A nationwide survey of a representative sample in Republic of Korea
Source: PLoS One. 2023 Jun 15;18(6):e0287182. doi: 10.1371/journal.pone.0287182 (PMC10270340; doi:10.1371/journal.pone.0287182)

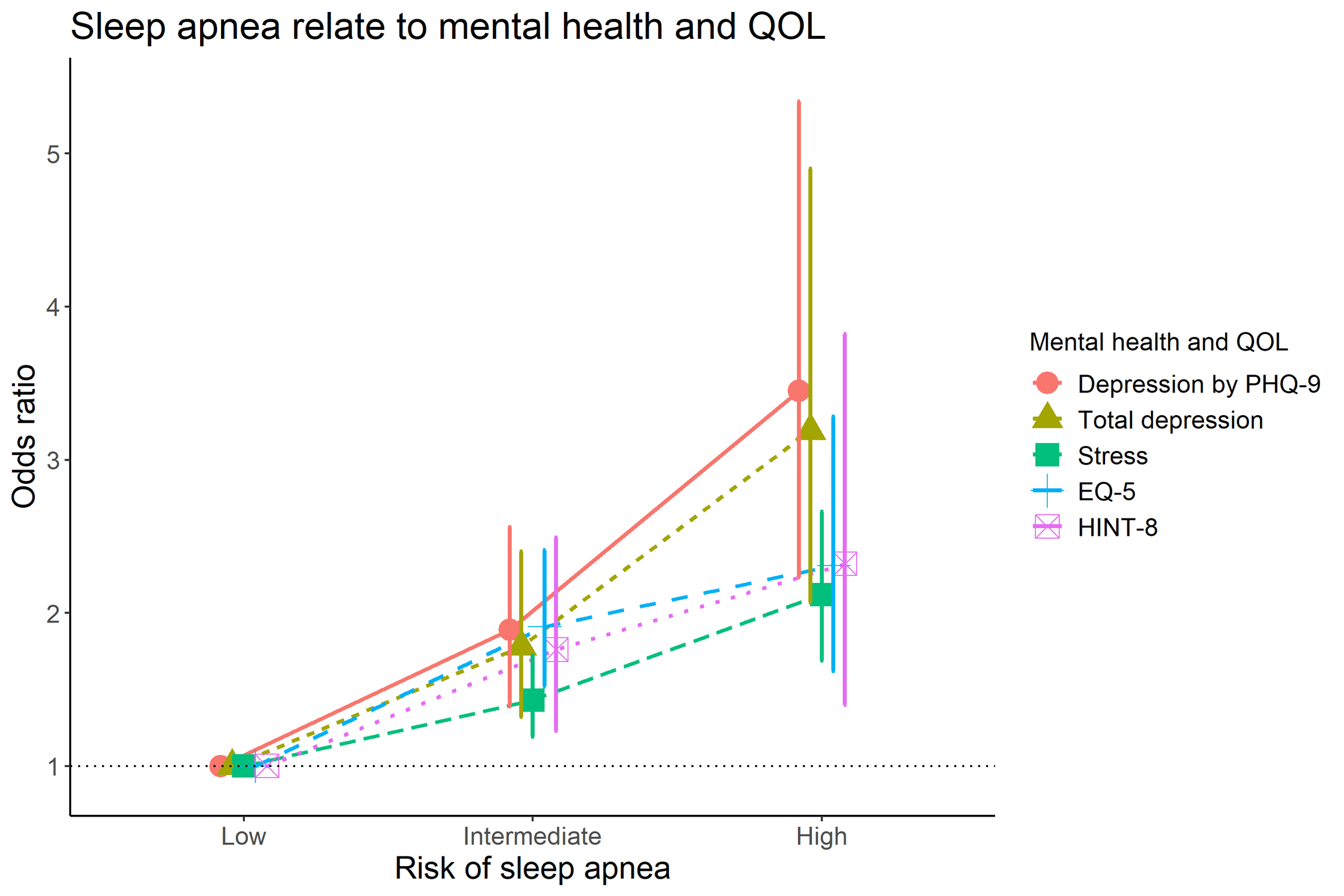

Supplement: S1 Fig — Adjusted for age, sex, education, alcohol consumption, smoking status, and physical activity, year, BMI, and multimorbidity. Abbreviations: QOL, quality of life; PHQ-9, Patient Health Questionnaire-9; EQ-5D, EuroQol Five-Dimension Questionnaire; HINT-8, Health-related Quality of Life Instrument with 8 Items. (TIF) [file pone.0287182.s001.tif]
